# Depletion of Essential Fatty Acids in the Food Source Affects Aerobic Capacities of the Golden Grey Mullet *Liza aurata* in a Warming Seawater Context

**DOI:** 10.1371/journal.pone.0126489

**Published:** 2015-06-01

**Authors:** Marie Vagner, Thomas Lacoue-Labarthe, José-Luis Zambonino Infante, David Mazurais, Emmanuel Dubillot, Hervé Le Delliou, Patrick Quazuguel, Christel Lefrançois

**Affiliations:** 1 UMR 7266 Littoral Environnement Sociétés, La Rochelle, France; 2 Ifremer, UMR 6539 Laboratoire des sciences de l’Environnement Marin, Plouzané, France; Sonoma State University, UNITED STATES

## Abstract

The objective of this study was to evaluate the combined effects of thermal acclimation and n-3 highly unsaturated fatty acids (n-3 HUFA) content of the food source on the aerobic capacities of fish in a thermal changing environment. The model used was the golden grey mullet *Liza aurata*, a species of high ecological importance in temperate coastal areas. For four months, fish were exposed to two food sources with contrasting n-3 HUFA contents (4.8% ecosapentaenoic acid EPA + docosahexaenoic acid DHA on the dry matter DM basis *vs.* 0.2% EPA+DHA on DM) combined with two acclimation temperatures (12°C *vs.* 20°C). The four experimental conditions were LH12, LH20, HH12 and HH20. Each group was then submitted to a thermal challenge consisting of successive exposures to five temperatures (9°C, 12°C, 16°C, 20°C, 24°C). At each temperature, the maximal and minimal metabolic rates, metabolic scope, and the maximum swimming speed were measured. Results showed that the cost of maintenance of basal metabolic activities was particularly higher when n-3 HUFA food content was low. Moreover, fish exposed to high acclimation temperature combined with a low n-3 HUFA dietary level (LH20) exhibited a higher aerobic scope, as well as a greater expenditure of energy to reach the same maximum swimming speed as other groups. This suggested a reduction of the amount of energy available to perform other physiological functions. This study is the first to show that the impact of lowering n-3 HUFA food content is exacerbated for fish previously acclimated to a warmer environment. It raises the question of the consequences of longer and warmer summers that have already been recorded and are still expected in temperate areas, as well as the pertinence of the lowering n-3 HUFA availability in the food web expected with global change, as a factor affecting marine organisms and communities.

## Introduction

Thermal tolerance varies widely among species and has been shown to be dependent on the acclimation temperature [1], as well as on the physiological plasticity of organisms [2]. In temperate latitudes, ectotherms often show a high physiological plasticity to thermal tolerance, which may greatly facilitate their adaptation to new environments and buffer the effects of daily or seasonal variations of environmental temperature on physiological processes [2].

When exposed to environmental temperature changes, ectotherms rapidly modulate the phospholipid composition of their cell membranes in terms of highly unsaturated fatty acids of the n-3 series (n-3 HUFA) in order to maintain their physiological performances [3,4]. This adjustment of the rate of n-3 HUFA in cell membrane determines the degree of unsaturation of the phospholipid and is necessary for maintaining membrane fluidity, and consequently the membrane functionality in a thermal changing environment [3]. For example, when the membrane fluidity tends to decrease at low temperatures, the degree of lipid unsaturation tends to increase to maintain its properties and functionality [5], and *vice versa* when the temperature increases [6]. These lipid adjustments in cell membranes can maintain the activity of transmembrane proteins [7,8], and consequently are determinant for the maintenance of numerous physiological functions (for review see [9–11]). In marine fish, membranous n-3 HUFA are mainly represented by ecosapentaenoic acid (EPA, 20:5n-3) and docosahexaenoic acid (DHA, 22:6n-3; for reviews see [9,11]). These fatty acids have been shown to be essential for growth, survival, pigmentation, development and functionality of the brain, vision, and nervous system, as well as in resistance to stress and disease, as they are precursors of eicosanoids (for reviews see [9,11]).

Although they function as critical structural and physiological components of the cell membranes of most tissues [9], n-3 HUFA are weakly synthesised *de novo* by higher organisms, and must be brought by food. In the natural food web, they are mainly supplied by marine microalgae, and especially by the dominating diatoms [12,13]. Several studies showed that n-3 HUFA present in the food source largely influenced swimming and metabolic performances that are essential to allow fish to migrate, feed, reproduce, or avoid predators [14–18]. However, these results are often conflicting, which may be related to the thermal environment of fish. For example, low levels of n-3 HUFA in the diet (replaced by a high rate of monounsaturated FA: oleic acid), and thus in cardiac tissue, led to higher swimming and cardiac performance of sea bass *Dicentrarchus labrax* acclimated to 20°C [16,17]. These authors suggested a more efficient oxidation of monounsaturated FAs when compared to polyunsaturated FAs in order to provide the necessary energy to the animal. A similar result was observed from a study performed at 9°C in Atlantic salmon [19,20]. However, a contrary result was measured in the same species tested at 10–12°C [18]. These different results for the same species could be due to the fact that the melting point of oleic acid is around 13°C, which would imply better membrane fluidity and thus modified physiological performance when the temperature is close to 13°C. These previous studies suggested some evidence of interactions between n-3 HUFA membrane content and thermal history on the physiology of marine organisms. However, little is known about the relative importance and potential interacting effects between temperature and n-3 HUFA food content on fish performance.

This investigation is of particular importance given the actual and predicted ecological context in temperate coastal areas. It is now known that summers are warmer and longer in these areas, and that an increase of 3°C in ocean water is predicted for the end of this century [21]. In the area of the Pertuis Charentais, located at the Atlantic mid-coast of France, and renowned for shellfish production and fisheries, this warming would lead to fish experiencing increasing seasonal temperature variations from ~8–20°C [22], to ~11–23°C [21]. Moreover, over the last twenty years, several studies have shown that modification of physicochemical parameters related to global change (*e*.*g*. increasing temperature, pH; decreasing salinity and oxygen saturation) led to a decreasing n-3 HUFA profile in diatoms, as well as to a decrease of their biomass in favor of more-adapted species such as cyanobacteria [23–26] (for review see [27]). This would lead to a lowering n-3 HUFA availability at the base of the food web, which may propagate to higher trophic levels, because, in all animals, it is well known that the fatty acid (FA) composition of the tissue is a reflection of their diet [28]. This would then lead to variability in the membranous n-3 HUFA content of higher consumers, despite the fact that membrane lipids are regulated to a large extent. Consequently, this would induce changes in membrane structure and function of higher consumers, which have been shown to further impact individual physiological performances.

In this context, the objective of this study was to evaluate the combined effect of thermal acclimation and n-3 HUFA content in food source on the membrane composition of the skeletal muscle and their potential consequences on physiological performance of the golden grey mullet, in terms of swimming and respiratory performances, in a thermal changing environment. The chosen species is of high ecological importance in the studied area as well as in European coastal areas because it is a microalgae grazer and a trophic vector of organic matter from intertidal to subtidal areas [29,30]. Because of its diet, the mullet may be directly subject to the variability of n-3 HUFA availability in primary producers, as no intermediate trophic steps will buffer their biochemical variability.

## Materials and Methods

To reach this objective, fish were exposed during four months to two contrasting n-3 HUFA dietary contents combined with two acclimation temperatures representative of mean winter and summer temperatures in the Pertuis Charentais. Then, fish were submitted to a thermal challenge consisting of successive exposures to five temperatures covering both seasonal changes of water temperature and the temperatures expected during the last of the century in the studied area [21,22]. At each temperature and for each fish, minimal and maximal oxygen consumption (MO_2min_ and MO_2max_ respectively), maximum swimming speed U_max_, and ventilation rate f_v_ were measured.

### Ethics statement

All fish manipulations were performed according to the Animal Care Committee of France (ACCF). No specific permissions were required from departmental service of fisheries to collect mullets *Liza aurata* in their natural environment, as this is not a protected or endangered species, and as few fish were collected (n = 160 individuals) regarding their abundance in this fishing area. The protocol was approved by the ACCF (approval number: 17-300-2). All fish manipulations were performed under anesthesia (tricaine methane sulphonate MS-222; 0.1 g L^-1^, Sigma-Aldrich, St Quentin-Fallavier, France), and all efforts were made to minimize suffering.

### Fish maintenance

Juvenile golden grey mullets [initial mean weight ± standard error (SE): 6.1 ± 0.2 g; initial mean standard length ± SE: 7.0 ± 0.1 cm] were caught in marine coastal area of La Rochelle (France) in 2012 and transported in aerated tank to our laboratory (Institut du Littoral et de l’Environnement, UMR 7266 CNRS-Université de La Rochelle, France) where all experiments were conducted. Upon arrival, the fish were transferred into four indoor tanks (volume: 400 L; n = 40 fish per tank) that were individually supplied with aerated recirculated sand-filtered natural seawater and equipped with an external biological filter (Eheim professional 3 2080, Eheim, Deizisau, Germany). They were progressively acclimated to the water tank temperature, which was kept constant by a recirculating water system (TECO TR20, Conselice, Italy), and maintained in a temperature-controlled room (20°C) exposed to a 12L:12D photoperiod cycle. Temperature (18.7 ± 0.04°C), salinity (29.3 ± 0.5) and oxygen (87.9 ± 2.6% air saturation) were monitored daily using a conductimeter (WTW model Oxi 340i, Weildeim, Germany). After a few days of acclimation to the experimental structure, each of the four initial tanks was separated in two tanks (n = 8 tanks in total with n = 20 fish per tank), and water temperature was progressively modified until 12°C (12.5 ± 0.1°C; n = 4 tanks), or 20°C (19.8 ± 0.1°C; n = 4 tanks). Fish were fed with a commercial diet (Le Gouessant aquaculture, Lamballe, France) once a day for three weeks.

Then, fish were progressively fed with two experimental isolipidic and isoproteic diets contrasting by their n-3 HUFA content, and made at the Ifremer-PFOM unit, UMR 6539 LEMAR (Plouzané, France): A high HUFA diet (HH: 4.8% EPA + DHA on dry matter basis), and a low-n-3 HUFA diet (LH: 0.2% EPA + DHA on dry matter basis DMB) which was obtained by replacing the fish oil present in the HH diet by soybean oil. For each of these two diets, 2 tanks per experimental temperature were fed for four months (2% of biomass day^-1^). Thus, the four experimental conditions were reared in duplicate and called HH20, LH20, HH12 and LH12. The diets were formulated taking a standard diet as a reference (1.2% EPA+DHA on DMB) that was previously formulated [15] according to the needs of other species, as the needs of *L*. *aurata* are not well known. The composition and FA content of the two experimental HH and LH diets are summarized in S1 Table.

### Lipid analysis

At the beginning of the experiment (T0), lipid analysis was performed on the muscle of 10 randomly sampled fish. After the four month experimental period, lipid analysis was performed on the muscle of 13 individuals randomly sampled for each experimental condition.

For each fish, whole frozen muscle was homogenized rapidly with a Hobart mixer in order to maintain a low temperature and then more accurately using a Polytron (PT 2100 Bioblock, Illkirch, France). A representative portion (~ 5g) was taken for lipid analysis and ~ 3g was taken for dry weight measurements (105°C in an oven for 24h). Lipid analysis was conducted on duplicates. Extraction of total lipids was performed automatically (Accelerated Solvent Extractor 350 Dionex, Fisher Scientific, Illkirch, France) according to the Folch method [31], with chloroform being replaced by dichloro-methane. The separation of neutral NL and polar lipids PL was performed on fish samples according to the procedure described by Juaneda and Roquelin [32]. The total lipids TL extracts were fractionated on silica cartridges (Chromabond SiOH, Macherey-Nagel, Hoerdt, France), NL were eluted by chloroform and PL by methanol. Fatty acid methyl esters (FAME) of TL, NL and PL were prepared by saponification and then methylation. All FAMEs were separated by gas chromatography (GC Clarus 500 Perkin-Elmer, Villebon-sur-Yvette, France) with a flame ionisation detector, BPX 70 capillary column: 25 m x 0.22 mm i.d. x 0.25μm film thickness; split-splitless injector, with helium as a carrier gas. The injector and detector temperatures were 220 and 260°C, respectively. The temperature of the oven was initially 50°C, and then increased to 180°C in increments of 15°C min^-1^; here, it was maintained for 5 min, and then finally increased to 220°C in increments of 3°C min^-1^. Data acquisition and handling were carried out by connecting the GC to a computer equipped with the TotalChrom workstation (Perkin-Elmer, Villebon-sur-Yvette, France). Individual FAMEs were identified by comparing the retention times of authentic standard mixtures. The results of individual FA composition were expressed as a percent of total identified FAME.

Chemical analyses of feed were performed in duplicate for each sample according to AOAC (Association of Official Analytical Chemists 1984) methods: ash (7 h at 550°C), crude fat [31], and crude protein (Dumas method with an Elementary NA 2000^®^, N × 6.25). FAME were extracted and analyzed on total lipid fraction using the previously described method.

### Growth performance

Just before being exposed to the two experimental LH and HH diets (T0), all 24h-starved fish were anaesthetized (MS-222; 0.1 g L^-1^, Sigma-Aldrich, St Quentin-Fallavier, France), weighed, and pit-tagged (MS-120; biolog-id, Réseaumatique, Bernay, France). Then, each month, 24h-starved fish (n = 40 per experimental condition) were anesthetized (MS-222; 0.1 g L^-1^, Sigma-Aldrich, St Quentin-Fallavier, France) and individually identified using a pit-tag reader (MS-120, biolog-id, Réseaumatique, Bernay, France) before measuring fresh weight (± 0.1 g), total length, standard length (*i*.*e*. notochord length), height and width (± 0.01 cm).

The specific growth rate SGR (% day ^-1^) was calculated for each experimental condition using Eq 1:

$$SGR=100\frac{\left(Ln_{\text{final}\;\text{body}\;\text{weight}}-Ln_{\text{initial}\;\text{body}\;\text{weight}}\right)}{\begin{array}{l} \text{Number}\;\text{of}\;\text{days} \end{array}}$$ (1)

The Fulton index (FI) representative of body condition was determined using Eq 2 [33]:

$$FI=100{\frac{W}{L^{3}}}^{\;}$$ (2)

where W is the fish weight in g and L the total fish length in cm.

### Thermal challenge

#### Experimental set-up

Fish metabolic and swimming performances were assessed during a thermal challenge using a swim-tunnel respirometer (Loligo Systems, Tjele, Denmark), which was made of a respirometer and an external bath. The respirometer (volume: 10 L) was composed of (i) a swim chamber with a square working section (40 cm of length, 10 cm height, 10 cm of width) and (ii) a hydraulic system placed upstream to promote a laminar flow in the swim chamber. No correction for solid blocking effects of the fish in the working section was made, since the calculated fractional error was < 5% of the working section area [34]. The flow in the respirometer was generated by an electric motor with a propeller. It was calibrated before the start of experiments and the speed ranged between 0 and 150 cm s^-1^. Temperature was kept constant by a recirculating water system from the external bath (Teco TR20, Conselice, Italy). A flush pump allowed water exchange between the respirometer and the external bath, in which water temperature and oxygenation were controlled.

#### Oxygen consumption measurements

Oxygen concentration in the respirometer was continuously measured during the experiments with an oxygen probe (PreSens, GmbH, Regensburg, Germany) connected to an oxymeter (Oxy-4, PreSens, GmbH, Regensburg, Germany) transferring oxygen data every 10 s to a storage computer. The oxygen concentration was automatically adjusted according to the real-time temperature recorded in the respirometer. Oxygen consumption (MO_2_) was measured by intermittent-flow respirometry, based on an alternation between (i) a flushing phase (5 min) and (ii) a measuring phase (20 min), during which the flush pump was turned off, preventing the inflow of water from the external bath into the respirometer. The MO_2_ (mgO_2_ kg^-1^ h^-1^) was calculated as in [15,35] (Eq 3):

$$MO_{2meas.}=\left(\frac{\Delta \;\left[\text{O}2\right]}{\Delta \text{t}\;}\right)\times \left(\frac{\text{V}}{\text{m}}\right)$$ (3)

where *Δ*[O_2_] is the oxygen concentration decrease (mgO_2_ L^-1^) relative to the fish oxygen consumption with respect to time *Δ*t (hours), V the swim tunnel water volume (10 L) minus the volume of the fish, and *m* the fish weight (kg).

For each MO_2_ measurement, a linear regression was adjusted (Graphical Analysis 3.4, Beaverton, OR, USA) in order to determine Δ[O_2_]/Δt from the graph plotting [O_2_] versus time. The regression coefficient of the linear relationship determined MO_2_ measurement accuracy. The bacterial MO_2_ was measured for half an hour before and after each experiment and at each temperature, and the mean was subtracted from the MO_2_ measured.

As respiratory metabolism depends on the animal weight, MO_2_ was standardized for a 100 g fish [35] (Eq 4):

$$MO_{2cor}{}_{.}=MO_{2meas.}\times \left(\frac{m_{meas}}{m_{cor}}\right){\;}^{1-A}$$ (4)

where MO_2cor_ (mgO_2_ kg^-1^ h^-1^) is the oxygen consumption for a corrected weight (m_cor_ = 100 g), MO_2meas_ is the measured MO_2_ (mgO_2_ kg^-1^ h^-1^) and m_meas_ is the fish weight (kg). A is the allometric exponent describing the relation between the metabolic rate and the fish weight. The A value has never been determined for *Liza aurata*. Therefore, we used a value of 0.8 which was previously estimated in teleosts [36], and already employed for *L*. *aurata* [15].

#### Experimental protocol of the thermal challenge

**Fig 1:**
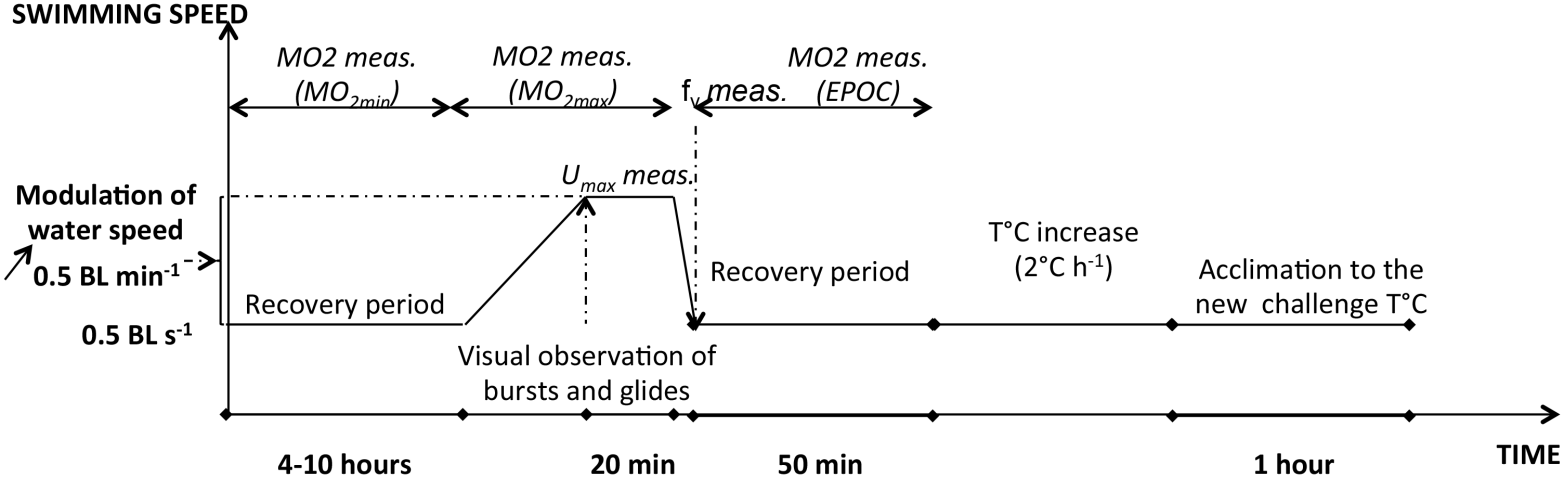
Diagram of the experimental protocol used for the thermal challenge. This diagram presents the evolution of swimming speed with time at one of the challenge temperature tested (*i*.*e*. 9°C, 12°C, 16°C, 20°C or 24°C). The different measures recorded for each fish and at each temperature are indicated in italic. MO_2min_: minimum oxygen consumption; MO_2max_: maximum oxygen consumption; U_max_: maximum swimming speed; f_v_: ventilation rate after the MO_2max_ challenge; EPOC: the excess post-oxygen consumption recorded during the recovery period following the swim challenge. The same protocol was repeated until each fish had been exposed to all challenge temperatures; one hour-acclimation to each new experimental challenge temperature was respected.

The experimental protocol used is presented in Fig 1.

Next, 96h-starved fish [HH20 group: mean weight ± SE: 39.6 ± 2.3 g; mean standard length ± SE: 13.3 ± 0.2 cm, n = 11; LH20 group: mean weight ± SE: 40.2± 2.2 g; mean standard length ± SE: 13.3 ± 0.2 cm; n = 11; HH12 group: mean weight ± SE: 14.9 ± 1.1 g; mean standard length ± SE: 9.5 ± 0.3 cm, n = 11; LH12 group: mean weight ± SE: 14.9 ± 1.4 g; mean standard length ± SE: 9.4 ± 0.2 cm; n = 11] were randomly sampled in tanks using a net, and individually tested in the swim-respirometer. This long starvation period ensured that swimming performance and oxygen consumption measurements were not influenced by digestion [37]. The day before the test, the fish were anesthetized (MS-222; 0.1 g L^-1^) and transferred into a plastic bag (without exposing the gills to the air, which may affect metabolism) from the indoor acclimatizing tank to the swim chamber of the swimming respirometer, in which the water temperature was similar to that of the rearing tank (*i*.*e*. 20°C or 12°C according to fish groups). After a short recovery period (about 5 min), the water flow was stabilized at a very low speed (0.5 Body Length BL s^-1^) and a screen darkening the upstream part of the swim chamber was placed in order to motivate the fish to occupy upstream positions. The fish were allowed to recover at this low speed for the next 4-10h during which MO_2_ was recorded to determine MO_2min_ [38]. The MO_2min_ was considered as the lowest MO_2_ value recorded during this period (Fig 1).

After this period, fish were submitted to an increasing swimming speed exercise to determine MO_2max_ (Fig 1). Swim tunnel was sealed from incoming oxygenated water from the external bath and MO_2_ measurements started. Water speed was progressively increased (0.5 BL min^-1^) until visual observation of the early stages of bursts and glide swimming suggesting that the fish was approaching its maximum aerobic swimming speed. This step was maintained for 20 min during which water speed was lightly modulated to ensure that fish was maximally challenged without being pushed to the grid at the rear of the swim tunnel. The MO_2max_ was considered the steepest slope of water oxygen saturation *vs*. time obtained during a 5 min period, and U_max_ (BL s^-1^) as the highest swimming speed reached during the test. Oxygen saturation never fell below 75% of air saturation during MO_2_ measurements. At the end of the 20 min-swimming test, water speed was progressively decreased to 0.5 BL s^-1^ and water of the respirometer was renewed through the flush pump reactivation during one minute, allowing the oxygen saturation to return over 85% of air saturation (Fig 1). Then, fish were allowed to recover for 50 min, during which MO_2_ was recorded according to a cycle of 20 min of MO_2_ measuring and 5 min of recirculating water, *i*.*e*. 2 MO_2_ measurements were recorded (Fig 1). These MO_2_ measurements allowed the estimation of the excess post-exercise oxygen consumption (EPOC) by integrating (Graphical Analysis vs. 3.4; Beaverton, OR, USA) the area between the post-test recovery MO_2_ curve and the MO_2min (0.5 BL s_^-1^_)_ measured before the swimming test [46]. In addition, gill ventilation frequency (f_v_; beats min^-1^) was video-recorded (Handycam DCR-HC23E, Sony) just after maximal swimming exercise. In order to get the highest ventilation rate, the first 15 s period was manually analyzed by counting opercula openings, by two experimenters and in duplicate.

At the end of the recovery period, water temperature was progressively changed in order to measure MO_2min_, MO_2max_, EPOC, and f_v_ at the five challenge temperatures: typically 9°C, 12°C, 16°C, 20°C and 24°C. Each new temperature was reached by increasing water temperature at a rate of ~2°C h^-1^ by regulating a heating system (Teco TR20, Conselice, Italy). Fish were given 1h to adjust to this new temperature (Fig 1). Then, they were held for 4-10h at this new temperature, and the protocol described above was repeated (Fig 1). In order to avoid too high temperature variations, the order at which challenge temperatures were tested depended on the temperature at which the fish were previously acclimated: Fish acclimated at 12°C were successively challenged at 12°C, 9°C, 16°C, 20°C, and 24°C, while fish reared at 20°C were successively challenged at 20°C, 24°C, 16°C, 12°C, and 9°C.

The fish were then removed from the swim tunnel, anesthetized (MS-222; 0.1 g L^-1^, Sigma-Aldrich, St Quentin-Fallavier, France), electronically identified, weighed and measured before being replaced in the initial tank.

### Data analyses

For each fish and at each temperature tested during the challenge, the aerobic scope AS and the net cost of transport at maximum exercise NCOT were calculated. AS (mgO_2_ kg^-1^ h^-1^) was determined for each fish as MO_2max_—MO_2min_ measured at the same temperature.

NCOT (mgO_2_ kg^-1^ m^-1^) was calculated according to Clark et al. [38]:

$$NCOT=\frac{\left(MO_{2\text{max}}-MO_{2\text{min}}\right)}{U_{\text{max}}}$$ (5)

### Statistical analysis

The data are presented as mean ± standard error (SE). The effects of diet and acclimation temperature on SGR and lipid content in fish muscles were tested using a two-way ANOVA with n = the number of fish replicates for each dietary condition Statistica software vs.7 (StatSoft, Maison-Alfort, France). Prior to ANOVA analyses, normality distribution and homeodasticity were controlled using Kolmogorov-Smirnov and the Levene test, respectively (Statistica software vs.7; StatSoft, Maison-Alfort, France). The effects of acclimation temperature, food (considered as a categorical covariate), challenge temperature (fixed effect), and factor interactions on ecophysiological variables (*i*.*e*. MO_2max_, MO_2min_, AS, U_max_, NCOT, EPOC and f_v max_) have been tested by applying multiple linear mixed effects models, where individual fish electronic identity has been considered a random factor. When covariate interactions were not significant, a simpler model was selected according to the Akaike Interaction Criterion (AIC). All models were computed using the *lme* fitting routine in R freeware (lme4 package; R Core Team, 2013). The level of significance for statistical analyses was always set at α = 0.05.

## Results

All groups of fish appeared healthy and survival rate was close to 100% at the end of the experiment.

### Fatty acid composition in fish

The initial and final FA compositions of fish are presented in S2 and S3 Tables, respectively.

As expected, final fish FA composition reflected that of their diet, and EPA (20:5n-3) and DHA (22:6n-3) were preferentially incorporated in PL than in NL for all fish groups (one-way ANOVA: EPA: *F*_*1*, *98*_ = 159.6; *P* < 0.001; DHA: *F*_*1*, *98*_ = 70.2; *P* < 0.001).

Regarding the initial fish composition, and as expected, the HH diet induced an increase in n-3 HUFA and Σ n-3 FA (in particular: 20:3n-3, 20:4n-3, 22:5n-3, and 22:6n-3 FA) in fish PL, while the LH diet induced a depletion in these FA contents, but an increase in n-6 FA instead.

In both lipid classes (PL and NL), Σ SFA, 22:6n-3 FA, Σ n-3 and Σ n-3 HUFA were higher in HH than in LH fish. EPA and the ratio n-3/n-6 FA contents were significantly affected by an interaction between acclimation temperature and diet, and EPA was less incorporated in PL and NL of LH20 fish. Concerning NL, 14:0, 16:0, 16:1, Σ MUFA, 18:2n-6, and Σ n-6 FA were significantly higher in HH fish, except for Σ n-6 and 18:2n-6, which were higher in LH fish. In PL, only 18:3n-3 was significantly affected by diet and was higher in LH than in HH fish.

FA integration in fish tissue also largely depends on acclimation temperature (S3 Table). In NL class, the 18:0 FA, Σ SFA, and the ratio DHA/EPA contents in fish were higher in fish reared at 12°C, while in PL class they were more efficiently incorporated in fish reared at 20°C. However, the opposite trend was observed for 14:0 FA. Concerning NL, 16:0, 18:2n-6, Σ n-6, 22:6n-3, Σ n-3 and Σ HUFA n-3 were preferentially incorporated in 12°C-acclimated than in 20°C-acclimated fish, except for the 16:0 FA, for which the contrary was observed. Concerning PL, the 20:0 FA, 16:1 FA, 18:3n-3 FA contents were higher in fish acclimated at 20°C than in those acclimated at 12°C.

Finally, an interactive effect of diet and acclimation temperature was observed for 18:3n-3 in NL, as for 18:2n-6 and Σ n-6 in PL class.

### Growth performances and body condition

**Fig 2:**
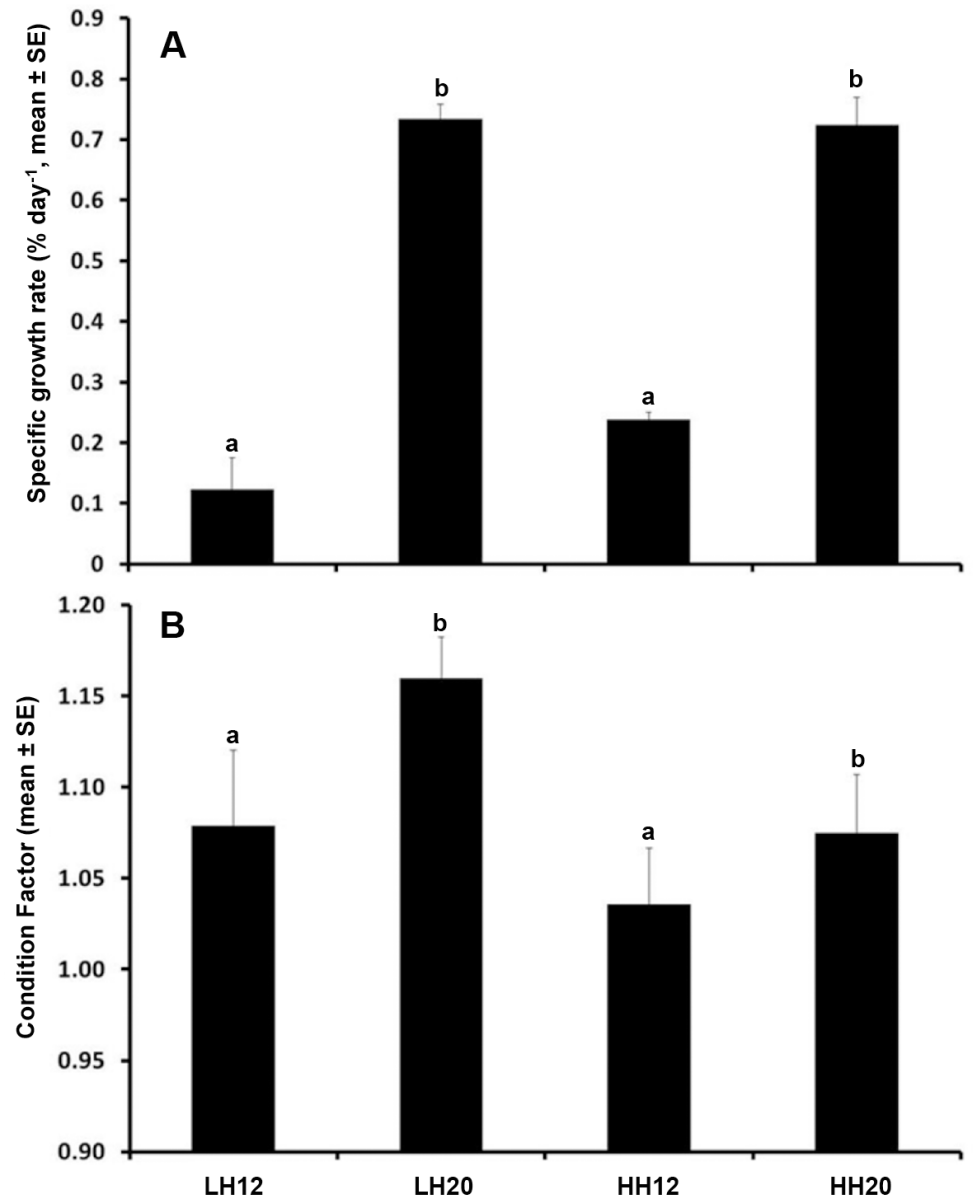
Combined effect of acclimation temperature and n-3 HUFA dietary content on growth performances. Specific growth rate SGR during the four months experimental period (% day^-1^; Fig A; LH12: n = 17; HH12: n = 37; LH20: n = 20; HH20: n = 34), and condition factor (Fulton Index FI) at the end of the experiment for fish which performed the thermal challenge (Fig B; n = 11 for each experimental fish group) for each experimental condition: high n-3 HUFA fed fish reared at 12°C (HH12) or 20°C (HH20), and low n-3 HUFA fed fish reared at 12°C (LH12) or 20°C (LH20). Bars with different letters are significantly different: for SGR: two-way ANOVA: effect of temperature: *F*_*1*,*103*_ = 1328.46; *P* < 0.001; effect of food: *F*_*1*,*103*_ = 1.917; *P* = 0.17; interaction food*temperature: *F*_*1*,*103*_ = 1.00; *P* = 0.32. For FI: two-way ANOVA: effect of temperature: *F*_*1*,*104*_ = 4.4; *P* = 0.037; effect of food: *F*_*1*,*3104*_ = 0.61; *P* = 0.44; interaction food*temperature: *F*_*1*,*104*_ = 3.8; *P* = 0.053).

As expected, the SGR and FI were significantly higher for fish reared at 20°C (Fig 2A and 2B). However, they were not affected by either the food, or by the interaction between food and acclimation temperature (P > 0.05).

### Swimming performances, energetics, and ventilation rate

The statistical results relative to MO_2max_, MO_2min_, AS, U_max_, NCOT, and f_v_ are reported in Table 1.

**Table 1:** Statistical effects of the environmental parameters tested on *Liza aurata* physiological performances.

Model	Parameter	*P*-value	AIC	Obs./Gr.
**MO**_**2max**_	Food	0.025*	1977	170 / 34
	Acc. temp.	0.024*		
	Chal. temp.	< 0.001***		
	Food x Acc. temp.	0.017*		
	Acc. temp. x Chal. temp.	0.013*		
**MO**_**2min**_	Food	0.025*	1581	175 / 35
	Acc. temp.	0.002**		
	Chal. temp.	< 0.001***		
**AS**	Food	0.182	1723	150 / 30
	Acc. temp.	0.008**		
	Chal. temp.	< 0.001***		
	Food x Acc. temp.	0.008**		
	Acc. temp. x Chal. temp.	< 0.001***		
**U**_**max**_	Food	0.328	604	185 / 27
	Acc. Temp.	< 0.001***		
	Chal. temp.	< 0.001***		
**NCOT**	Food	0.058.	- 585	110 / 22
	Acc. temp.	0.003**		
	Chal. temp.	0.002**		
	Food x Acc. Temp.	0.032*		
	Acc. temp. x Chal. temp.	< 0.001***		
	Food x Cond T x Chal. temp.	0.005**		
**EPOC**	Food	0.817	1882	146 / 38
	Acc. Temp.	< 0.001***		
	Chal. temp.	< 0.001***		
	Acc. temp. x Chal. temp.	< 0.001***		
**f**_**v**_	Food	0.179	1710	185 / 38
	Acc. temp.	< 0.001***		
	Chal. temp.	< 0.001***		

Parameters of linear mixed effect models applied to the maximal oxygen consumption MO_2max_, the minimal oxygen consumption MO_2min_, the aerobic scope AS, the maximal swimming speed U_max_, the net cost of transport NCOT, the excess post-exercise oxygen consumption EPOC and the ventilation rate after swimming effort f_v_ as a function of the challenge temperature. Only significant interactions between factors tested are reported in this Table. Abbreviations: Acc. temp.: acclimation temperature; AIC: Akaike Interaction Criterion; Chal. temp.: challenge temperature; Gr.: number of fish; Obs.: number of observations.

**Fig 3:**
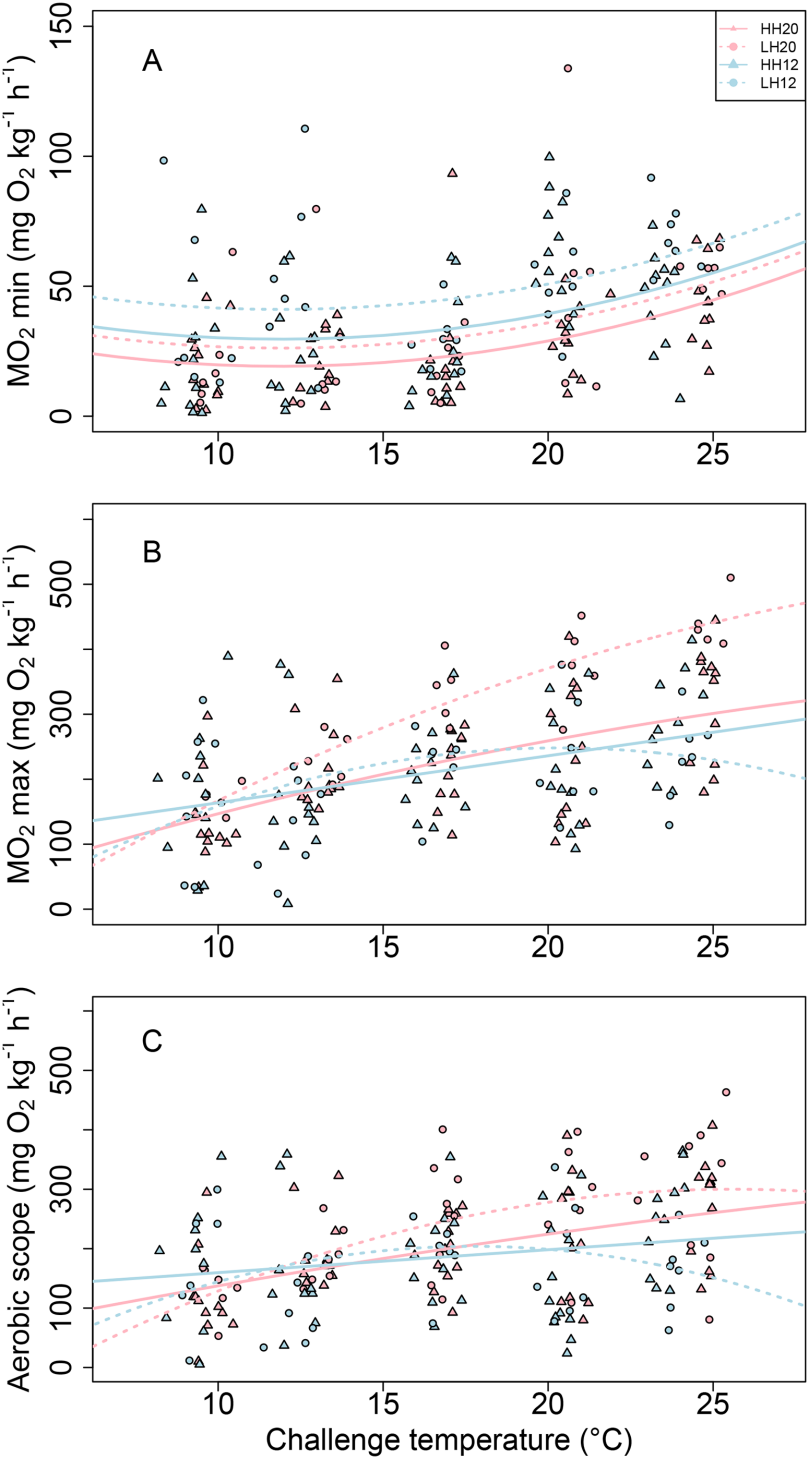
Combined effect of acclimation temperature and n-3 HUFA dietary content on aerobic metabolism. (A) Minimal (MO_2 min_) and (B) maximal oxygen consumption rate (MO_2 max_) and (C) aerobic scope of fish acclimated at 20°C (dark symbols) and 12°C (light symbols) and fed with enriched (triangle; HH) or depleted (circle symbol; LH) n-3 HUFA regime as a function of the challenge temperature over a range from 9°C to 24°C. Regression lines are described by polynomial models of equation: AxChalTemp^2^ + BxChalTemp + C, where ChalTemp is the challenge temperature tested. Statistical results are reported in Table 1.

MO_2min_ varied similarly throughout the thermal challenge in all experimental fish groups: it decreased slightly from 9°C to 12°C, and was minimal at this temperature, and then increased until 24°C (Table 1; Fig 3A). However, regardless of the challenge temperature or the acclimation temperature, MO_2min_ was 20% lower for the HH fish than for LH. It was also almost 30% higher for the 12°C-conditioned fish than for the 20°C-conditioned fish in both dietary groups (Fig 3A).

MO_2max_ was significantly affected by an interactive effect between acclimation temperature and challenge temperature (Table 1; Fig 3B). Fish acclimated at 20°C showed an increasing MO_2max_ all along the challenge but did not reach their maximal rate within the tested range of challenge temperatures. Among them, the LH20 fish displayed a higher increase of MO_2max_ with challenge temperature than the HH20 (Table 1), reaching an MO_2max_ value 30% higher than them at 24°C. Among the fish conditioned at 12°C, the LH12 displayed its highest MO_2max_ at 20°C (232.1 ± 33.3 mg O_2_ kg^-1^ h^-1^), while the HH12 fish displayed a slight but continuous increase of MO_2max_ with challenge temperature. These fish displayed their highest MO_2_ below 10°C (Fig 3), but their MO_2max_ values did not exceed those of the HH20 fish at 24°C.

As for MO_2max_, AS was significantly affected by an interactive effect between acclimation and challenge temperature, as well as by an interactive effect between acclimation temperature and diet (Table 1; Fig 3C). Globally, AS of the LH12 fish increased from 9°C to 17.7°C (until 215.0 ± 30.0 mg O_2_ kg^-1^ h^-1^), while LH20 fish tented to reach a maximum at 24°C (290.4 ± 44.7 mg O_2_ kg^-1^ h^-1^). In contrast, HH12 and HH20 fish kept increasing their maximum AS when exposed to successive challenge temperatures (236.2 ± 24.8 mg O_2_ kg^-1^ h^-1^ and 268.6 ± 25.1 mg O_2_ kg^-1^ h^-1^ at 24°C respectively).

**Fig 4:**
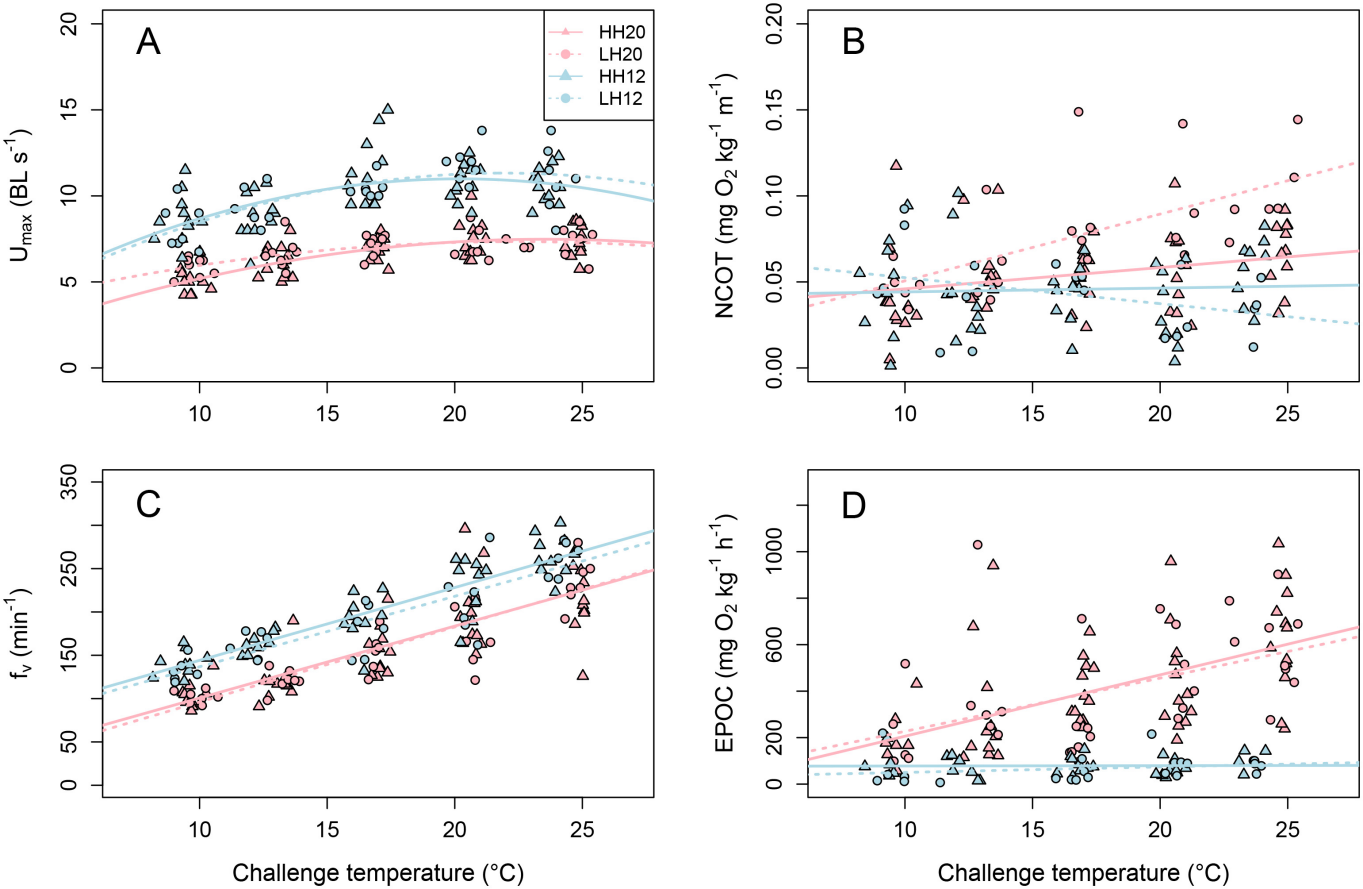
Combined effect of acclimation temperature and n-3 HUFA dietary content on metabolic performances. (A) Maximal swimming speed (U_max_), (B) net cost of transport at maximum exercise (NCOT), (C) post-challenge ventilation rate (f_v_) and (D) excess post-exercise oxygen consumption (EPOC) of fish acclimated at 20°C (dark points) and 12°C (light points) and fed with enriched (triangle symbol; HH) or depleted (circle symbol; LH) n-3 HUFA regime for four months as a function of the challenge temperature along a range from 9°C to 24°C. Regression lines for U_max_ are described by polynomial models of the equation: AxChalTemp^2^ + BxChalTemp + C, where ChalTemp is the challenge temperature tested. Statistical results are reported in Table 1.

U_max_ was significantly affected by acclimation temperature but not by diet (Table 1; Fig 4A). Regardless of the challenge temperature, U_max_ was about 40% higher in 12°C-acclimated fish than in 20°C-acclimated group. Moreover, U_max_ varied similarly all along the thermal challenge at both acclimation temperatures, increasing when challenge temperature increased from 9°C to 20°C, and remaining stable when challenge temperature increased from 20°C to 24°C: U_max_ varied from 5.4 ± 0.2 at 9°C to 7.4 ± 0.2 BL s^-1^ at 24°C and from 8.6 ± 0.3 at 9°C to 10.8 ± 0.3 at 24°C BL s^-1^ for 20°C- and 12°C-acclimated fish, respectively.

The NCOT was stable throughout the challenge concerning 12°C-acclimated fish (from 0.05 ± 0.01 mg O_2_ kg^-1^ m^-1^ at 9°C to 0.05 ± 0.01 mg O_2_ kg^-1^ m^-1^ at 24°C; Table 1; Fig 4B). However, concerning 20°C-acclimated fish, NCOT progressively increased throughout the thermal challenge, and became progressively significantly higher in LH than in HH fish. This difference between dietary fish groups varied from 10% at 12°C, to 30% at 24°C.

As expected, the f_v_ significantly increased with challenge temperature in all fish groups, due to the increasing metabolic demand, as shown by the global MO_2max_ increase with challenge temperature, combined to the decreasing oxygen availability and blood oxygen affinity with increasing temperature [39,40] (Table 1; Fig 4C). The f_v_ was not affected by diet, but was 10% to 26% higher for the 12°C-acclimated fish than for the 20°C-acclimated fish throughout the thermal challenge (Table 1; Fig 4C).

### Excess post-exercise oxygen consumption (EPOC)

The EPOC was not affected by diet, and was almost stable throughout the thermal challenge concerning the 12°C-acclimated fish, ranging from 63.7 ± 39.2 and 89.5 ± 28.7 mg O_2_ kg^-1^ h^-1^ at 9°C, to 82.2 ± 10.5 and 106.1 ± 24.4 mg O_2_ kg^-1^ h^-1^ at 24°C for the LH and the HH fish, respectively (Table 1; Fig 4D). However, it significantly increased with the challenge temperature in the 20°C-acclimated fish, and was from two- to six-fold higher than in the 12°C-acclimated fish from 9°C to 24°C.

## Discussion

To the best of our knowledge, the present study is the first to highlight the combined effects of n-3 HUFA content in the food source and acclimation temperature on the swimming and metabolic performances of *Liza aurata* in a thermal changing environment. While several studies reported the effects of n-3 HUFA on fish performance, this work revealed that these effects on fish aerobic capacities are highly dependent on acclimation temperature. These results are of particular interest in the context of fish response to the natural or induced spatio-temporal variations of temperature and HUFA availability occurring in coastal areas.

This study aimed to use highly contrasting diets in terms of n-3 HUFA content, in order to observe highly contrasted physiological responses of fish. The n-3 HUFA content of the LH diet (0.2% EPA+DHA on DM) was about 24-fold lower than that of the HH diet (4.8% EPA+DHA on DM). Compared to the standard diet previously used for this species [15] and established from the needs known for other species (~1% EPA+DHA on DM), the LH diet was considered deficient, while the HH diet was considered rich in n-3 HUFA. The immediate consequence of feeding fish with both of these diets was the modification of the composition of the cell membrane bilayer in both LH and HH fish [41], [42], as (1) fish fed the LH diet presented the lowest n-3 HUFA content, including EPA and DHA, and (2) both of these fatty acids were preferentially incorporated in PL rather than NL in both LH and HH fish, validating their integration in cell membranes [41].

First, clear differences in aerobic capacities have been highlighted with respect to the acclimation temperature. Compared to 12°C-acclimated fish the 20°C-acclimated fish 1) spent much more energy (NCOT) to reach a lower swimming speed value (U_max_) during effort and 2) showed a higher EPOC, revealing a higher oxygen debt during the recovery period following swimming effort. This higher EPOC indicates that the 20°C-acclimated fish relied more on anaerobic metabolism during the swim challenge than the 12°C-acclimated fish [43–45]. This means that the ionic, osmotic and biochemical imbalances in metabolites such as lactate and glycogen [43] were associated with swimming effort, which induced a higher MO_2_ to restore tissue and cellular energy balance after the swim challenge. However, this higher EPOC was not correlated with a higher f_v_ during the recovery period [47–49], confirming that 20°C-acclimated fish have a limited capacity to increase oxygen supply at the systemic level compared to 12°C-acclimated fish for which higher f_v_ combined with lower EPOC suggested a better capacity to recover from the test, regardless of the challenge temperature. This higher anaerobic metabolism utilization linked to a lower swimming performance may be partly explained by the lower number or diameter of oxidative fibers within the myotome, as observed following acclimation to warm waters in mosquitofish [50], goldfish [51] or striped bass [52]. This reduced oxidative area would have then prevented swimming to be supported by aerobic muscular activity up to higher velocities, involving the recruitment of the fast anaerobic fibers.

Second, the basal metabolic cost of all fish groups increased when challenge temperature was above 15°C, implying that seawater warming may increase the cost of the maintenance of basal activities such as cardiac pump or osmoregulation for *Liza aurata* [53]. Moreover, for both acclimation temperatures tested, the LH fish displayed higher MO_2min_ regardless of the challenge temperature, suggesting that more energy is necessary to maintain vital functions when fish are exposed to deficient n-3 HUFA food sources. This is congruent with a previous result from McKenzie [14], who found that sturgeons and eels fed a rich n-3 HUFA diet had significantly lower basal metabolic rate than fish fed a depleted n-3 HUFA diet. These congruent results in different species indicate that dietary fatty acids can have very similar effects on metabolism in widely different taxonomic fish groups, irrespective of their thermal acclimation or temperature exposure.

The LH fish, when conditioned at 20°C (LH20), displayed a higher aerobic metabolism (showed by higher MO_2max_ and AS) than the other fish, supporting a higher capacity for oxygen allocation towards energy-demanding activities such as swimming, foraging, growth and/or digestion [53]. Nevertheless, these fish spent much more energy (higher NCOT) than HH20 to reach a similar maximum swimming speed (U_max_), and this was even more important as the challenge temperature increased. Taken together, these results imply that the supplementary oxygen quantity available for LH20 is much more rapidly consumed to perform the same level of activity as the HH20 fish. This revealed an altered yield between oxygen-consumption and muscular work caused by depleted n-3 HUFA supply. The higher energy spent by LH20 fish to swim at the same velocity may lead to less excess energy to perform other energy-demanding activities. This is expected to be disadvantageous for mullets in a natural environment, since they have to perform concomitantly several energy-consuming actions such as migration, foraging, or predator escape. In the long-term, this little excess energy could lead to a reduction of the specific growth rate, as the excess aerobic energy is expected to be allocated to growth once other activities have been allocated [53]. Such a reduction in growth rate has not been observed in this study, based on 4 months of acclimation, but has been previously reported in the same species following 5-months of acclimation at 20°C with the LH diet [15]. It is noteworthy that, in our experimental conditions, the fish did not have to use their aerobic metabolism to forage (food provided in sufficient quantity) or to escape from predators. This implies that, in a natural environment where fish have to perform these other activities at the same time as swimming, an alteration of energy metabolism due to n-3 HUFA deficiency could more rapidly impact on their ecophysiological performances such as growth. Longer experimental work combined with fieldwork testing fish in their more restrictive natural environment would be valuable to validate this hypothesis and assess the time lapse for significant impact.

Thus, this study highlighted that LH fish displayed higher metabolic rates than HH fish, with higher MO_2max_ and AS when these LH fish were acclimated at 20°C than at 12°C. This could be due to several mechanisms. First, EPA was the only FA content interactively affected by acclimation temperature and diet: it was less incorporated in the PL and NL when fish were acclimated at 20°C and fed the LH diet. Thus, the lipid composition seemed to have responded to increasing temperature with a decreasing degree of unsaturation [6–8]. Moreover, high acclimation temperature reduced the content of 18:3n-3 FA in PL, an important EPA precursor, reducing the possibility of compensating for EPA decrease by *de novo* production. Consequently, the lower EPA incorporation measured in the LH20 fish could have modified membrane functionality, consequently impacting the physiological performances of fish. It is worth noting that, in addition, this lower EPA incorporation in LH20 fish could lead to an alteration of their immune functions, as the EPA is a precursor of eicosanoids used to produce leukotrienes, thromboxanes and prostaglandins (for reviews see [12–14]). A second hypothesis would be a combined effect of low n-3 HUFA content in the food source and high acclimation temperature on proton leak process, which is defined as an increasing mitochondrial MO_2_ without increasing ATP production [54,55]. This MO_2_ is controlled by inner membrane permeability to protons. On one hand, the diet may have increased mitochondrial proton leak through a modification of permeability of the mitochondrial membranes. In mammals, n-3 HUFA deficiency in membrane has been previously shown to increase mitochondrial proton leak [56]. This theory may explain the higher MO_2max_ but the similar U_max_ reached by the LH fish in comparison to the HH fish. On the other hand, proton leak may have been increased by warm-acclimation temperature [57]. Uncoupling proteins, capable of increasing proton leakage across the inner mitochondrial membrane and then reducing the aerobic ATP formation of cells, have been found in several fish species [57–60], and their level has been shown to be higher in warm-acclimated Antarctic eelpout *Pachycara brachycephalum* [57]. This suggested a lower ATP production combined to a higher MO_2_ in these fish than in cold-acclimated group. A third hypothesis could be the higher linoleic acid concentration in the LH diet, known to increase carnitine palmitoyl transferase activity, which may improve the aerobic metabolism of FAs in red muscle [61,62]. Previous works suggested that aerobic metabolism is primarily fuelled by FA oxidation, with MUFA being preferred over SFAs, which, in turn, are preferred over HUFA as substrates [61–63]. However, this last hypothesis is not supported by our results, as MUFA or SFA contents in the muscle of LH fish were lower than in HH fish.

This study brings a set of new clues showing that the impact of a trophic source depleted in n-3 HUFA is exacerbated for fish acclimated to a warmer environment, directly impacting several physiological functions that all depend on metabolic capacities, and in the long-term may reduce ecophysiological performance, such as growth. In a natural environment, these results raise the question of the ecological consequences of longer and warmer summers already recorded and still expected in temperate areas [21]. Indeed, warmer events led to a decreasing n-3 HUFA profile in diatoms, as well as to a decrease of their biomass in favor of the development of poor-HUFA cyanobacteria (for review see [27]), exposing mullets to environmental conditions that could interactively challenge their metabolism. Beyond this seasonal aspect, where fish could respond through phenotypic plasticity or relocation, this work again underlines the pertinence of the change of trophic source quality, especially within the context of the global warming, as a factor affecting living organisms and marine communities.

## Supporting Information

S1 Table Formulation and fatty acid composition in total lipids of the two experimental diets. Fatty acids for which the percentage was lower than 0.2% FAME are not represented. Each MUFA is represented as a sum of n-7, n-9 and n-11 FA. ^a^ Sources: fish meal LT 94: Norse (Fyllingsdalen, Norway); casein: Sigma-Aldrich (Germany); soy oil: Système U (Créteil, France); fish oil: pure cod oil Cooper (Melun, France); precooked starch: Prégéflo Roquette frères (Lestrem, France); vitamin mixture (INRA Jouy-en-Josas, France). ^b^ Vitamin mixture (g kg^-1^ vitamin mix): retinyl acetate, 1; cholecalciferol, 2 5; DL-α-tocopheryl acetate, 5; menadione, 1; thiamine-HCL, 0 1; riboflavin, 0 4; D-calcium panththenate, 2; pyridoxine-HCL, 0 3; cyanocobalamin, 1; niacin, 1; choline, 200; ascorbic acid (ascorbyl polyphosphate), 5; folic acid, 0 1; D-biotin, 1; meso-inositol, 30. ^c^ Mineral mixture (g kg^-1^ mineral mix): KCl, 90; KI, 0 04; CaHPO_4_ 2H_2_O, 500; NaCl, 40; CuSO_4_ 5H_2_O, 3; ZnSO_4_ 7H_2_O, 4; CoSO_4_, 0 02; FeSO_4_ 7H_2_O, 20; MnSO_4_ H_2_O, 3; CaCo_3_, 215; MgOH, 124; Na_2_SeO_3_, 0 03; NaF, 1. Abbreviations: ARA: arachidonic acid; DHA docosahexaenoic acid; EPA: ecosapentaenoic acid; FA: fatty acids; HH: high n-3 HUFA diet; HUFA: highly unsaturated fatty acids; LH: low-n-3 HUFA diet; MUFA: mono-unsaturated fatty acids; SE: standard error; SFA: saturated fatty acids; TL: total lipids.
(PDF)

S2 Table Fatty acid composition of *Liza aurata* muscle before the experiment (T0). Total lipid TL content, neutral lipid NL content, polar lipid PL content (mg g^-1^ of dry weight), fatty acid profile of NL and PL (% of fatty acids methyl esters FAME) in *Liza aurata* white muscle at the T0 of the experiment (n = 10). Values are mean ± standard error. Abbreviations: ARA: arachidonic acid; DHA docosahexaenoic acid; EPA: ecosapentaenoic acid; HUFA: highly unsaturated fatty acids; MUFA: mono-unsaturated fatty acids; SFA: saturated fatty acids.
(PDF)

S3 Table Fatty acid composition of *Liza aurata* muscle at the end of the experiment. Total lipid TL content, neutral lipid NL content, polar lipid PL content (mg g^-1^ of dry weight), fatty acid profile of NL and PL (% of fatty acids methyl esters FAME) in *Liza aurata* white muscle at the end of the experiment according to rearing conditions (HH20: fish fed the high-n-3 HUFA diet and reared at 20°C, n = 14; LH20: fish fed the low-n-3 HUFA diet and reared at 20°C, n = 13; HH12: fish fed the high-n-3 HUFA diet and reared at 12°C, n = 13; and LH12: fish fed the low-n-3 HUFA diet and reared at 12°C, n = 10). Values are mean ± standard error. Statistical significance of diet (D), temperature (T), as well as the interaction between both factors (D*T) are indicated through the *P*, *F* and *df* values (two-way ANOVA). Significance was considered from α < 0.05 and was indicated in bold case in the table. Values containing different letters on a same row are significantly different. Abbreviations: ARA: arachidonic acid; D: diet; D*T: interaction between diet and temperature; *df*_*1*_: degree of freedom of numerator; *df*_*2*_: degree of freedom of denominator; DHA docosahexaenoic acid; EPA: ecosapentaenoic acid; HUFA: highly unsaturated fatty acids; MUFA: mono-unsaturated fatty acids; SFA: saturated fatty acids; T: temperature.
(PDF)
