# Supplementary material for: Depletion of Essential Fatty Acids in the Food Source Affects Aerobic Capacities of the Golden Grey Mullet Liza aurata in a Warming Seawater Context
Source: PLoS One. 2015 Jun 1;10(6):e0126489. doi: 10.1371/journal.pone.0126489 (PMC4452649; doi:10.1371/journal.pone.0126489)
Supplement: S1 Table — Fatty acids for which the percentage was lower than 0.2% FAME are not represented. Each MUFA is represented as a sum of n-7, n-9 and n-11 FA. a Sources: fish meal LT 94: Norse (Fyllingsdalen, Norway); casein: Sigma-Aldrich (Germany); soy oil: Système U (Créteil, France); fish oil: pure cod oil Cooper (Melun, France); precooked starch: Prégéflo Roquette frères (Lestrem, France); vitamin mixture (INRA Jouy-en-Josas, France). b Vitamin mixture (g kg-1 vitamin mix): retinyl acetate, 1; cholecalciferol, 2 5; DL-α-tocopheryl acetate, 5; menadione, 1; thiamine-HCL, 0 1; riboflavin, 0 4; D-calcium panththenate, 2; pyridoxine-HCL, 0 3; cyanocobalamin, 1; niacin, 1; choline, 200; ascorbic acid (ascorbyl polyphosphate), 5; folic acid, 0 1; D-biotin, 1; meso-inositol, 30. c Mineral mixture (g kg-1 mineral mix): KCl, 90; KI, 0 04; CaHPO4 2H2O, 500; NaCl, 40; CuSO4 5H2O, 3; ZnSO4 7H2O, 4; CoSO4, 0 02; FeSO4 7H2O, 20; MnSO4 H2O, 3; CaCo3, 215; MgOH, 124; Na2SeO3, 0 03; NaF, 1. Abbreviations: ARA: arachidonic acid; DHA docosahexaenoic acid; EPA: ecosapentaenoic acid; FA: fatty acids; HH: high n-3 HUFA diet; HUFA: highly unsaturated fatty acids; LH: low-n-3 HUFA diet; MUFA: mono-unsaturated fatty acids; SE: standard error; SFA: saturated fatty acids; TL: total lipids. (PDF) [file pone.0126489.s001.pdf]

S1 Table. Formulation and fatty acid composition in total lipids of the two experimental diets.

**Ingredients [a] (g 100 g^{-1})**	**HH diet**	**LH diet**
Fish meal LT 94	17	17
Casein	30	30
Soy oil	0	10
Fish oil	12	0
Precooked starch	30	30
Vitamin mixture [b]	7	7
Mineral mixture [c]	3	3
Betaine	1	1
**Chemical composition (% dry matter DM ± SE)**		
Crude protein (% DM)	13.3 ± 0.3	13.2 ± 0.2
Crude fat (% DM)	35.4 ± 0.5	34.6 ± 0.5
Dry matter (%)	93.1 ± 0.1	91.2 ± 0.1
Ash (% DM)	7.1 ± 0.1	7.6 ± 0.4
HUFA n-3 (% DM)	4.8 ± 0.3	0.2 ± 0.0
**FA composition in TL (% fatty acid methyl ester FAME ± SE)**		
14:0	3.83 ± 0.04	0.6 ± 0.04
16:0	13.21 ± 0.01	11.05 ± 0.12
18:0	3.17 ± 0.05	4.20 ± 0.04
20:0	0.31 ± 0.01	0.40 ± 0.00
Σ SFA	20.73 ± 0.09	16.58 ± 0.18
16:1	4.26 ± 0.05	0.72 ± 0.03
18:1	34.52 ± 0.07	31.94 ± 0.13
20:1	4.75 ± 1.39	1.39 ± 0.01
22:1	3.26 ± 0.14	1.01 ± 0.01
24:1	0.16 ± 0.01	0.07 ± 0.00
ΣMUFA	42.56 ± 0.34	34.47 ± 0.13
18:2n-6	14.77 ± 0.2	39.67 ± 0.30
20:4n-6	0.3 ± 0.00	0.08 ± 0.01
Σ n-6	15.91 ± 0.195	39.75 ± 0.28
18:3n-3	3.34 ± 0.09	6.02 ± 0.07
18:4n-3	0.77 ± 0.04	0.15 ± 0.00
20:4n-3	0.59 ± 0.01	0.07 ± 0.00
20:5n-3	3.27 ± 0.08	0.59 ± 0.01
22:5n-3	0.81 ± 0.01	0.08 ± 0.01
22:6n-3	2.92 ± 0.03	0.80 ± 0.00
Σ n-3	11.89 ± 0.24	7.70 ± 0.08
Σ HUFA n-3	7.79 ± 0.12	1.53 ± 0.01
n-3 / n-6	0.74 ± 0.01	0.19 ± 0.00
DHA / EPA	0.89 ± 0.01	1.34 ± 0.01
ARA / EPA	0.09 ± 0.00	0.12 ± 0.00

Fatty acids for which percentage was lower than 0.2% FAME are not represented. Each MUFA is represented as a sum of n-7, n-9 and n-11 FA.

[a] Sources: fish meal LT 94: Norse (Fyllingsdalen, Norway); casein: Sigma-Aldrich (Germany); soy oil: Système U (Créteil, France); fish oil: pure cod oil Cooper (Melun, France);

precooked starch: Prégéflo Roquette frères (Lestrem, France); vitamin mixture (INRA Jouy-en-Josas, France)

[b] Vitamin mixture (g kg^{-1} vitamin mix): retinyl acetate, 1; cholecalciferol, 2 5; DL-α-tocopheryl acetate, 5; menadione, 1; thiamine-HCL, 0 1; riboflavin, 0 4; D-calcium panththenate, 2; pyridoxine-HCL, 0 3; cyanocobalamin, 1; niacin, 1; choline, 200; ascorbic acid (ascorbyl polyphosphate), 5; folic acid, 0 1; D-biotin, 1; meso-inositol, 30

[c] Mineral mixture (g kg^{-1} mineral mix): KCl, 90; KI, 0 04; $CaHPO_4$ $2H_2O$, 500; NaCl, 40; $CuSO_4$ $5H_2O$, 3; $ZnSO_4$ $7H_2O$, 4; $CoSO_4$, 0 02; $FeSO_4$ $7H_2O$, 20; $MnSO_4$ H_2O, 3; $CaCo_3$, 215; MgOH, 124; Na_2SeO_3, 0 03; NaF, 1

Abbreviations: ARA: arachidonic acid; DHA docosahexaenoic acid; EPA: ecosapentaenoic acid; FA: fatty acids; HH: high n-3 HUFA diet; HUFA: highly unsaturated fatty acids; LH: low-n-3 HUFA diet; MUFA: mono-unsaturated fatty acids; SE: standard error; SFA: saturated fatty acids; TL: total lipids.
