# Supplementary material for: Depletion of Essential Fatty Acids in the Food Source Affects Aerobic Capacities of the Golden Grey Mullet Liza aurata in a Warming Seawater Context
Source: PLoS One. 2015 Jun 1;10(6):e0126489. doi: 10.1371/journal.pone.0126489 (PMC4452649; doi:10.1371/journal.pone.0126489)
Supplement: S2 Table — Total lipid TL content, neutral lipid NL content, polar lipid PL content (mg g-1 of dry weight), fatty acid profile of NL and PL (% of fatty acids methyl esters FAME) in Liza aurata white muscle at the T0 of the experiment (n = 10). Values are mean ± standard error. Abbreviations: ARA: arachidonic acid; DHA docosahexaenoic acid; EPA: ecosapentaenoic acid; HUFA: highly unsaturated fatty acids; MUFA: mono-unsaturated fatty acids; SFA: saturated fatty acids. (PDF) [file pone.0126489.s002.pdf]

S2 Table. Fatty acid composition of *Liza aurata* muscle before the experiment (T0).

	T0	
**TL**	21.86 ± 1.92	
**NL**	6.70 ± 1.14	
**PL**	13.97 ± 2.27	
	**PL**	**NL**
**14:0**	5.25 ± 0.19	3.39 ± 0.12
**16:0**	19.58 ± 0.48	22.59 ± 1.86
**18:0**	3.04 ± 0.16	4.09 ± 1.11
**20:0**	0.24 ± 0.01	0.32 ± 0.02
**Σ SFA**	28.39 ± 0.55	30.12 ± 2.52
**16:1**	9.01 ± 0.56	7.26 ± 0.74
**18:1**	16.65 ± 0.66	21.34 ± 0.99
**Σ MUFA**	29.04 ± 0.80	35.44 ± 1.32
**18:2n-6**	9.73 ± 0.18	14.08 ± 0.87
**20:4n-6**	0.77 ± 0.09	0.50 ± 0.04
**Σ n-6**	14.51 ± 0.70	14.96 ± 0.95
**18:3n-3**	1.47 ± 0.07	1.20 ± 0.09
**20:3n-3**	0.11 ± 0.01	0.12 ± 0.01
**20:4n-3**	0.52 ± 0.02	0.45 ± 0.02
**20:5n-3**	7.15 ± 0.61	3.97 ± 0.46
**22:5n-3**	1.47 ± 0.05	1.30 ± 0.05
**22:6n-3**	4.29 ± 0.19	5.52 ± 0.49
**Σ n-3**	16.99 ± 0.78	12.69 ± 0.63
**Σ HUFA n-3**	13.53 ± 0.64	10.91 ± 0.39
**n-3 / n-6**	1.21 ± 0.10	0.88 ± 0.07
**DHA / EPA**	0.65 ± 0.07	1.70 ± 0.38
**ARA / EPA**	0.11 ± 0.01	0.12 ± 0.01

Total lipid TL content, neutral lipid NL content, polar lipid PL content (mg g^{-1} of dry weight), fatty acid profile of NL and PL (% of fatty acids methyl esters FAME) in *Liza aurata* white muscle at the T0 of the experiment (n = 10). Values are mean ± standard error.

Abbreviations: ARA: arachidonic acid; DHA docosahexaenoic acid; EPA: ecosapentaenoic acid; HUFA: highly unsaturated fatty acids; MUFA: mono-unsaturated fatty acids; SFA: saturated fatty acids.
