# Supplementary material for: Depletion of Essential Fatty Acids in the Food Source Affects Aerobic Capacities of the Golden Grey Mullet Liza aurata in a Warming Seawater Context
Source: PLoS One. 2015 Jun 1;10(6):e0126489. doi: 10.1371/journal.pone.0126489 (PMC4452649; doi:10.1371/journal.pone.0126489)
Supplement: S3 Table — Total lipid TL content, neutral lipid NL content, polar lipid PL content (mg g-1 of dry weight), fatty acid profile of NL and PL (% of fatty acids methyl esters FAME) in Liza aurata white muscle at the end of the experiment according to rearing conditions (HH20: fish fed the high-n-3 HUFA diet and reared at 20°C, n = 14; LH20: fish fed the low-n-3 HUFA diet and reared at 20°C, n = 13; HH12: fish fed the high-n-3 HUFA diet and reared at 12°C, n = 13; and LH12: fish fed the low-n-3 HUFA diet and reared at 12°C, n = 10). Values are mean ± standard error. Statistical significance of diet (D), temperature (T), as well as the interaction between both factors (D*T) are indicated through the P, F and df values (two-way ANOVA). Significance was considered from α < 0.05 and was indicated in bold case in the table. Values containing different letters on a same row are significantly different. Abbreviations: ARA: arachidonic acid; D: diet; D*T: interaction between diet and temperature; df 1: degree of freedom of numerator; df 2: degree of freedom of denominator; DHA docosahexaenoic acid; EPA: ecosapentaenoic acid; HUFA: highly unsaturated fatty acids; MUFA: mono-unsaturated fatty acids; SFA: saturated fatty acids; T: temperature. (PDF) [file pone.0126489.s003.pdf]

S3 Table. Fatty acid composition of *Liza aurata* muscle at the end of the experiment.

	Fish composition				Statistical analysis				
	HH20	LH20	HH12	LH12	*Effect*	*P*	*F*	df_1	df_2
**TL**	18.16 ± 0.84	20.57 ± 1.52	17.93 ± 2.06	15.87 ± 1.32	D	0.909	0.93	1	46
					T	0.117	2.55	1	46
					D*T	0.154	2.09	1	46
**NL**	13.13 ± 0.77	14.41 ± 1.20	7.57 ± 1.55	8.05 ± 1.25	D	0.482	0.50	1	46
					**T**	**< 0.001**	**23.12**	**1**	**46**
					D*T	0.751	0.10	1	46
**PL**	3.57 ± 0.34[a]	4.70 ± 0.51[ab]	8.16 ± 0.62[c]	6.52 ± 0.62[bc]	D	0.630	0.24	1	46
					**T**	**< 0.001**	**36.79**	**1**	**46**
					**D*T**	**0.012**	**6.83**	**1**	**46**
**NL**									
**14:0**	3.88 ± 0.10	3.03 ± 0.09	3.45 ± 0.15	2.74 ± 0.20	**D**	**<0.001**	**33.77**	**1**	**46**
					**T**	**0.011**	**7.063**	**1**	**46**
					D*T	0.595	0.286	1	46
**16:0**	23.29 ± 0.21	21.20 ± 0.25	17.92 ± 0.91	16.89 ± 0.66	**D**	**0.009**	**7.371**	**1**	**46**
					**T**	**< 0.001**	**70.99**	**1**	**46**
					D*T	0.358	0.861	1	46
**18:0**	1.79 ± 0.02	1.86 ± 0.06	2.48 ± 0.34	2.07 ± 0.11	D	0.373	0.81	1	46
					**T**	**0.020**	**5.80**	**1**	**46**
					D*T	0.200	1.69	1	46
**20:0**	0.19 ± 0.00	0.20 ± 0.01	0.21 ± 0.02	0.19 ± 0.02	D	0.345	0.912	1	46
					T	0.345	0.909	1	46
					D*T	0.344	0.914	1	46
**Σ SFA**	29.34 ± 0.27	26.43 ± 0.28	24.24 ± 1.26	22.09 ± 0.79	**D**	**0.001**	**11.01**	**1**	**46**
					**T**	**< 0.001**	**38.17**	**1**	**46**
					D*T	0.621	0.247	1	46
**16:1**	9.00 ± 0.14	7.02 ± 0.15	6.99 ± 0.27	6.32 ± 0.50	**D**	**< 0.001**	**24.11**	**1**	**46**
					**T**	**< 0.001**	**25.42**	**1**	**46**
					**D*T**	**0.019**	**5.86**	**1**	**46**
**18:1**	32.45 ± 0.33	31.71 ± 0.35	30.89 ± 0.59	30.64 ± 0.62	D	0.131	2.36	1	46
					**T**	**0.005**	**6.275**	**1**	**46**

					D*T	0.671	0.183	1	46
**Σ MUFA**	46.25 ± 0.33	41.21 ± 0.35	44.08 ± 0.99	41.08 ± 0.92	**D**	**< 0.001**	**33.97**	**1**	**46**
					T	0.104	2.76	1	46
					D*T	0.145	2.19	1	46
**18:2n-6**	9.73 ± 0.18	21.22 ± 0.47	13.88 ± 0.60	22.33 ± 1.87	**D**	**< 0.001**	**135.86**	**1**	**46**
					**T**	**0.003**	**9.46**	**1**	**46**
					D*T	0.081	3.19	1	46
**20:4n-6 (AA)**	0.24 ± 0.02	0.11 ± 0.01	0.30 ± 0.01	0.20 ± 0.02	D	0.332	0.96	1	46
					T	0.337	0.94	1	46
					D*T	0.347	0.90	1	46
**Σ n-6**	10.57 ± 0.19	21.72 ± 0.47	15.01 ± 0.57	23.27 ± 1.86	**D**	**< 0.001**	**132.70**	**1**	**46**
					**T**	**< 0.001**	**12.63**	**1**	**46**
					D*T	0.094	2.92	1	46
**18:3n-3**	1.85 ± 0.04[a]	2.81 ± 0.06[b]	1.96 ± 0.13[ac]	2.37 ± 0.22[bc]	**D**	**< 0.001**	**33.24**	**1**	**46**
					T	0.162	2.02	1	46
					**D*T**	**0.024**	**5.41**	**1**	**46**
**20:3n-3**	0.16 ± 0.00	0.10 ± 0.00	0.22 ± 0.01	0.18 ± 0.01	D	0.16	2.00	1	46
					T	1.16	2.01	1	46
					D*T	0.16	1.97	1	46
**20:4n-3**	0.40 ± 0.01	0.12 ± 0.01	0.44 ± 0.03	0.21 ± 0.02	D	0.32	1.03	1	46
					T	0.34	0.94	1	46
					D*T	0.35	0.89	1	46
**20:5n-3 (EPA)**	2.10 ± 0.07[c]	0.87 ± 0.10[a]	2.20 ± 0.14[c]	1.51 ± 0.21[b]	**D**	**< 0.001**	**54.73**	**1**	**46**
					**T**	**0.006**	**8.27**	**1**	**46**
					**D*T**	**0.046**	**4.17**	**1**	**46**
**22:5n-3**	0.94 ± 0.02 [c]	0.28 ± 0.01[a]	1.21 ± 0.06 [d]	0.62 ± 0.06 [b]	**D**	**< 0.001**	**275.72**	**1**	**46**
					**T**	**< 0.001**	**71.38**	**1**	**46**
					**D*T**	**< 0.001**	**7.90**	**1**	**46**
**22:6n-3 (DHA)**	2.56 ± 0.07	0.86 ± 0.05	3.76 ± 0.26	2.26 ± 0.39	**D**	**< 0.001**	**56.87**	**1**	**46**
					**T**	**< 0.001**	**37.67**	**1**	**46**
					D*T	0.642	0.22	1	46
**Σ n-3**	8.62 ± 0.14	5.37 ± 0.16	10.34 ± 0.34	7.60 ± 0.39	**D**	**< 0.001**	**127.55**	**1**	**46**
					**T**	**< 0.001**	**55.55**	**1**	**46**
					D*T	0.337	0.94	1	46
**Σ HUFA n-3**	6.15 ± 0.12	2.24 ± 0.14	7.76 ± 0.32	4.79 ± 0.46	**D**	**< 0.001**	**165.15**	**1**	**46**

					**T**	**< 0.001**	**60.48**	**1**	**46**
					D*T	0.084	3.13	1	46
**n-3 / n-6**	0.82 ± 0.01[b]	0.25 ± 0.01[a]	0.70 ± 0.03[b]	0.37 ± 0.06[a]	**D**	**< 0.001**	**196.67**	**1**	**46**
					T	0.974	0.01	1	46
					**D*T**	**< 0.001**	**0.17**	**1**	**46**
**DHA / EPA**	1.24 ± 0.05	1.11 ± 0.12	1.84 ± 0.21	1.82 ± 0.46	D	0.175	1.90	1	46
					**T**	**0.011**	**7.05**	**1**	**46**
					D*T	0.883	0.02	1	46
**ARA / EPA**	0.11 ± 0.01	0.13 ± 0.01	0.14 ± 0.01	0.14 ± 0.02	D	0.247	1.37	1	46
					T	0.053	3.94	1	46
					D*T	0.404	0.71	1	46
**PL**									
**14:0**	3.01 ± 0.49	2.84 ± 0.42	4.60 ± 0.17	3.78 ± 0.14	D	0.190	1.77	1	46
					**T**	**0.001**	**11.61**	**1**	**46**
					D*T	0.389	0.76	1	46
**16:0**	18.37 ± 1.04	17.55 ± 1.04	16.79 ± 0.31	16.72 ± 0.40	D	0.595	0.29	1	46
					T	0.157	2.07	1	46
					D*T	0.655	0.20	1	46
**18:0**	7.75 ± 1.02	6.23 ± 0.83	3.79 ± 0.12	3.98 ± 0.30	D	0.369	0.82	1	46
					**T**	**< 0.001**	**17.81**	**1**	**46**
					D*T	0.250	1.36	1	46
**20:0**	0.27 ± 0.02	0.23 ± 0.02	0.18 ± 0.01	0.17 ± 0.01	D	0.170	1.94	1	46
					**T**	**< 0.001**	**18.21**	**1**	**46**
					D*T	0.322	1.00	1	46
**ΣSFA**	29.95 ± 0.85	27.19 ± 0.77	25.71 ± 0.37	24.99 ± 0.60	**D**	**0.016**	**6.21**	**1**	**46**
					**T**	**< 0.001**	**21.27**	**1**	**46**
					D*T	0.167	1.97	1	46
**16:1**	5.82 ± 0.82	5.57 ± 0.61	7.48 ± 0.27	6.54 ± 0.28	D	0.32	1.01	1	46
					**T**	**0.032**	**4.91**	**1**	**46**
					D*T	0.563	0.34	1	46
**18:1**	20.89 ± 0.86	22.47 ± 0.74	21.57 ± 0.52	20.49 ± 0.55	D	0.725	0.13	1	46
					T	0.364	0.84	1	46
					D*T	0.069	3.46	1	46
**ΣMUFA**	29.99 ± 1.54	29.82 ± 1.19	32.53 ± 0.68	29.02 ± 0.69	D	0.120	2.51	1	46
					T	0.458	0.56	1	46

					D*T	0.158	2.06	1	46
**18:2n-6**	7.35 ± 0.36^{a}	19.18 ± 0.60^{c}	11.50 ± 0.49^{b}	19.44 ± 1.50^{c}	**D**	**< 0.001**	**169.92**	**1**	**46**
					**T**	**< 0.001**	**14.906**	**1**	**46**
					**D*T**	**< 0.001**	**17.36**	**1**	**46**
**20:4n-6 (AA)**	1.54 ± 0.19	0.97 ± 0.17	1.01 ± 0.07	0.84 ± 0.08	**D**	**0.010**	**7.20**	**1**	**46**
					T	0.077	3.27	1	46
					D*T	0.196	1.72	1	46
**Σ n-6**	9.73 ± 0.26^{a}	21.11 ± 0.45^{c}	13.43 ± 0.46^{b}	21.16 ± 1.43^{c}	**D**	**< 0.001**	**208.18**	**1**	**46**
					**T**	**< 0.001**	**12.63**	**1**	**46**
					**D*T**	**0.010**	**16.43**	**1**	**46**
**18:3n-3**	1.58 ± 0.16	2.89 ± 0.23	2.45 ± 0.12	3.19 ± 0.22	**D**	**< 0.001**	**30.85**	**1**	**46**
					**T**	**0.002**	**9.91**	**1**	**46**
					D*T	0.123	2.47	1	46
**20:3n-3**	0.25 ± 0.02	0.22 ± 0.02	0.25 ± 0.01	0.26 ± 0.01	D	0.37	0.83	1	46
					T	0.23	1.47	1	46
					D*T	0.24	1.45	1	46
**20:4n-3**	0.65 ± 0.03	0.23 ± 0.02	0.69 ± 0.02	0.37 ± 0.05	D	0.34	0.94	1	46
					T	0.40	0.74	1	46
					D*T	0.37	0.80	1	46
**20:5n-3 (EPA)**	5.81 ± 0.40^{b}	3.08 ± 0.34^{a}	6.03 ± 0.27^{b}	4.70 ± 0.30^{b}	**D**	**< 0.001**	**35.02**	**1**	**46**
					**T**	**0.01**	**7.13**	**1**	**46**
					**D*T**	**0.047**	**4.14**	**1**	**46**
**22:5n-3**	2.97 ± 0.30	1.56 ± 0.25	2.38 ± 0.08	1.70 ± 0.11	**D**	**< 0.01**	**21.84**	**1**	**46**
					T	0.31	1.05	1	46
					D*T	0.11	2.68	1	46
**22:6n-3 (DHA)**	11.85 ± 1.55	6.28 ± 1.07	7.06 ± 0.38	5.68 ± 0.69	**D**	**0.002**	**10.68**	**1**	**46**
					T	0.062	3.65	1	46
					D*T	0.095	2.90	1	46
**Σ n-3**	23.65 ± 2.06	14.64 ± 1.44	20.06 ± 0.57	16.75 ± 0.89	**D**	**< 0.001**	**17.57**	**1**	**46**
					T	0.617	0.253	1	46
					D*T	0.059	3.76	1	46
**Σ HUFA n-3**	21.49 ± 2.26	11.37 ± 1.68	16.41 ± 0.62	12.70 ± 1.01	**D**	**< 0.001**	**17.75**	**1**	**46**
					T	0.258	1.31	1	46
					D*T	0.057	3.81	1	46
**n-3 / n-6**	2.47 ± 0.23^{a}	0.70 ± 0.08^{c}	1.52 ± 0.08^{b}	0.87 ± 0.14^{c}	**D**	**< 0.001**	**91.82**	**1**	**46**

					**T**	**0.016**	**6.17**	**1**	**46**
					**D*T**	**0.002**	**10.55**	**1**	**46**
**DHA / EPA**	1.94 ± 0.17	1.87 ± 0.16	1.19 ± 0.08	1.21 ± 0.12	D	0.878	0.02	1	46
					**T**	**< 0.001**	**24.26**	**1**	**46**
					D*T	0.921	0.01	1	46
**ARA / EPA**	0.26 ± 0.02	0.29 ± 0.02	0.17 ± 0.01	0.18 ± 0.01	D	0.115	2.577	1	46
					**T**	**< 0.001**	**22.28**	**1**	**46**
					D*T	0.891	0.019	1	46

Total lipid TL content, neutral lipid NL content, polar lipid PL content (mg g^{-1} of dry weight), fatty acid profile of NL and PL (% of fatty acids methyl esters FAME) in *Liza aurata* white muscle at the end of the experiment according to rearing conditions (HH20: fish fed the high-n-3 HUFA diet and reared at 20°C, n = 14; LH20: fish fed the low-n-3 HUFA diet and reared at 20°C, n = 13; HH12: fish fed the high-n-3 HUFA diet and reared at 12°C, n = 13; and LH12: fish fed the low-n-3 HUFA diet and reared at 12°C, n = 10). Values are mean ± standard error. Statistical significance of diet (D), temperature (T), as well as the interaction between both factors (D*T) are indicated through the *P*, *F* and *df* values (two-way ANOVA). Significance was considered from α < 0.05 and was indicated in bold case in the table. Values containing different letters on a same row are significantly different.

Abbreviations: ARA: arachidonic acid; D: diet; D*T: interaction between diet and temperature; df_1: degree of freedom of numerator; df_2: degree of freedom of denominator; DHA docosahexaenoic acid; EPA: ecosapentaenoic acid; HUFA: highly unsaturated fatty acids; MUFA: mono-unsaturated fatty acids; SFA: saturated fatty acids; T: temperature.
